# Simultaneous detection and differentiation of canine parvovirus and feline parvovirus by high resolution melting analysis

**DOI:** 10.1186/s12917-019-1898-5

**Published:** 2019-05-10

**Authors:** Yaru Sun, Yuening Cheng, Peng Lin, Hewei Zhang, Li Yi, Mingwei Tong, Zhigang Cao, Shuang Li, Shipeng Cheng, Jianke Wang

**Affiliations:** 1 Key Laboratory of Special Animal Epidemic Disease, Ministry of Agriculture, No. 4899, Juye Street, Jingyue District, Changchun, 130112 People’s Republic of China; 2 Institute of Special Animal and Plant Sciences, Chinese Academy of Agricultural Sciences, No. 4899, Juye Street, Jingyue District, Changchun, 130112 People’s Republic of China

**Keywords:** Simultaneous detection, Differentiation, Canine parvovirus, Feline parvovirus, HRM

## Abstract

**Background:**

Canine parvovirus (CPV) and feline parvovirus (FPV) are causative agents of diarrhea in dogs and cats, which manifests as depression, vomiting, fever, loss of appetite, leucopenia, and diarrhea in young animals. CPV and FPV can single or mixed infect cats and cause disease. To diagnose sick animals effectively, an effective virus diagnostic and genome typing method with high sensitivity and specificity is required.

**Results:**

In this study, a conserved segment containing one SNP A4408C of parvovirus was used for real-time PCR amplification. Subsequently, data were auto-analyzed and plotted using Applied Biosystems® High Resolution Melt Software v3.1. Results showed that CPV and FPV can be detected simultaneously in a single PCR reaction. No cross-reactions were observed with canine adenovirus, canine coronavirus, and canine distemper virus. The assay had a detection limit of 4.2 genome copies of CPV and FPV. A total of 80 clinical samples were subjected to this assay, as well as to conventional PCR-sequence assay and virus isolation. Results showed that the percentage of agreement of the assay and other methods are high.

**Conclusions:**

In short, we have developed a diagnostic test for the accurate detection and differentiation of CPV and FPV in fecal samples, which is also cost effective.

## Background

Parvoviruses are linear, non-segmented single-stranded DNA viruses, with an average genome size of about 5000 nucleotides. *Parvoviridae* is divided into two subfamilies, *Parvovirinae* and *Densovirinae*, which infect vertebrate and insects, respectively, and cause a wide range of diseases in insects, animals, and humans [1, 2]. *Parvovirinae* was further divided into eight genera, including *Protoparvovirus* and so on [3]. Canine parvovirus (CPV) and feline parvovirus (FPV) are potentially fatal pathogens for domestic dogs and cats as well as for various wild species, characterized by vomiting, fever, leucopenia, and diarrhea in carnivores, especially life threatening for young animals up to the age of 6 months.

FPV was first isolated from a sick cat in 1965 [4]. Thirteen years later, a variant of an FPV like virus named CPV was identified in the fecal samples of dogs with diarrhea and spread worldwide rapidly [5]. Since then, the original CPV-2, which cannot infect cats, was subsequently replaced by three different but closely related antigenic variants (CPV-2a, CPV-2b, and CPV-2c), which can infect cats [5, 6]. CPV has evolved more rapidly than FPV, the substitution rate for the CPV and FPV clade was 1.7 × 10^− 4^ and 9.4 × 10^− 5^ substitutions per site per year, respectively [7].

Recently, it was reported that 80% of parvovirus isolates from domestic and leopard cats in Vietnam and Taiwan were CPV [8]. Compared with cases that 32.5 and 33.9% infection rates of CPV were demonstrated in a feline-only shelter and a mixed canine and feline shelter in the UK, respectively [8, 9]. In addition, Battilani et al. reported a co-infection case, which was identified to be infected by a new parvovirus variant inter CPV and FPV [10]. Furthermore, studies revealed the inter-FPV subspecies recombination and inter-antigenic recombination of CPV in vaccinated pups were demonstrated, which elucidates a novel mechanism for the emergence of CPV and the evolution of parvoviruses in nature, instead of the high and positive selection theory of parvovirus evolution [11, 12]. Thus, it’s important to develop a diagnostic method that can detect and differentiate CPV from FPV in the same sample. However, traditional methods, such as DNA sequence and hemagglutination inhibition (HI) assay using a panel of monoclonal antibodies, are expensive, labor-intensive and time-consuming [13, 14]. Afterward, taking into account a single polymorphism G3752A of the viral genome (reference strain: CPV Laika-1993 GenBank accession no. JN033694), a set of primers and probes, FPV/CPV-For, FPV/CPV-Rev and FPV/CPV-Pbs (VIC and FAM), was designed to develop a TaqMan MGB real-time PCR method for differentiating CPV from FPV [15]. However, this TaqMan MGB real-time PCR method was not used for all the prevalent isolates in China, because there are variants of CPV with nucleotide mutant at site 3752, e.g. the HB3 isolate, with the nucleotide mutant of A3752G, (GenBank accession no. GU392238) (Zhang, L. and Yan, X. J., unpublished data). With the application of saturated fluorescent dye, fluorescence data are collected by qPCR instruments at finer temperature resolution and the data are processed in the intuitive software platforms. High resolution melting analysis (HRM) assay is becoming a new method to quickly, inexpensively and efficiently scan clinical samples [16–18].

In the study, we developed an HRM assay by taking advantages of the more conserved segment and one SNP detection and use it to distinguish CPV from FPV in clinical samples simultaneously.

## Results

### Standard plasmid preparation

**Fig. 1:**
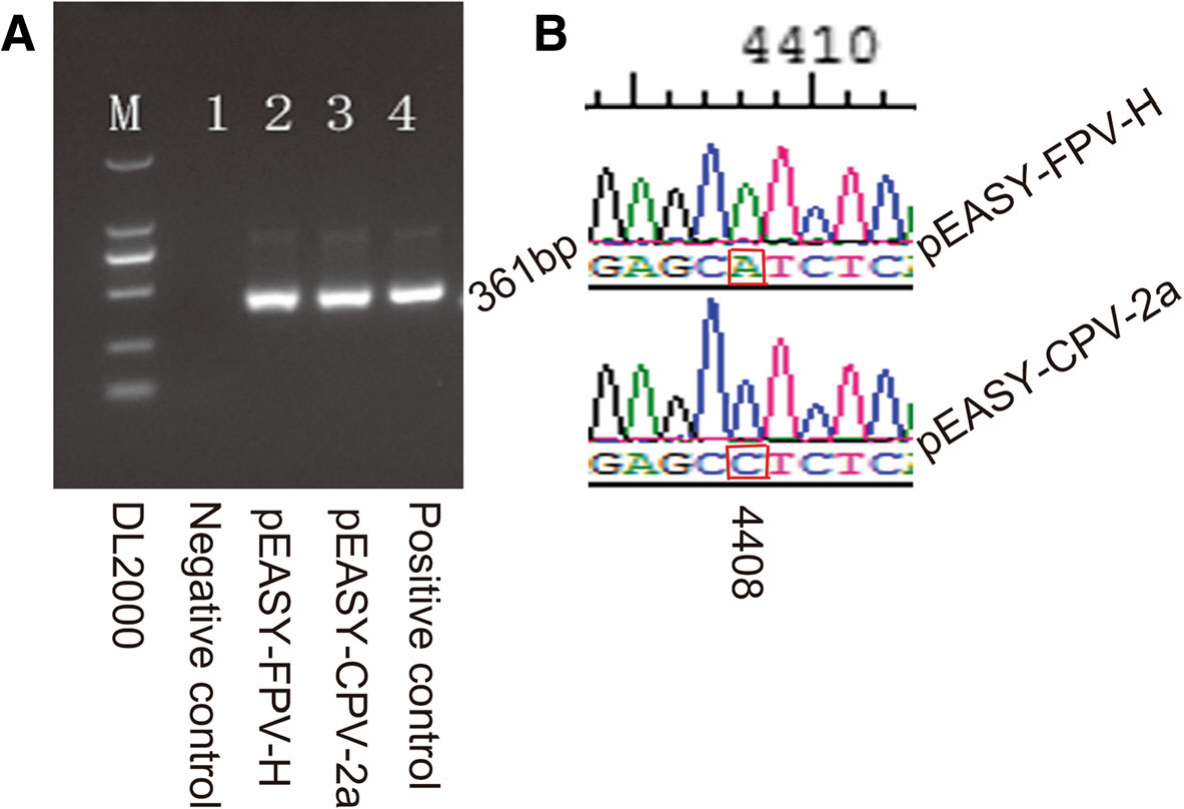
Determination of recombinant plasmids pEASY-FPV-H and pEASY-CPV-2a. The two plasmids were PCR from CPV JL14–1 strain and vaccine FPV with primers F1 and R1 and cloned to pEASY™-T5 zero vector, and then identified by PCR and sequencing. **a** Agarose gel (3%) showing the amplification of recombinant plasmids from FPV and CPV. Lane M: DL2000 (Takara Biotechnology Co., Ltd., Dalian, China); lane 1: Negative control; lane 2: pEASY-FPV-H; lane 3: pEASY-CPV-2a; lane 4: Positive DNA of CPV JL14–1 strain. **b** Alignment the sequences of the two plasmids. The SNP at the relevant position of pEASY-FPV-H and pEASY-CPV-2a are same to the original viruses, FPV, and CPV (A and C, respectively)

The recombinant plasmids were verified by PCR and sequencing (Fig. 1). A 361 bp fragment was amplified from pEASY-FPV-H and pEASY-CPV-2a by PCR with the primer pair F1/R1 (Fig. 1a). Subsequently, the single SNP used in the design of the HRM assay was detected when amplified products were sequenced and aligned (Fig. 1b).

### qPCR and HRM analysis

**Fig. 2:**
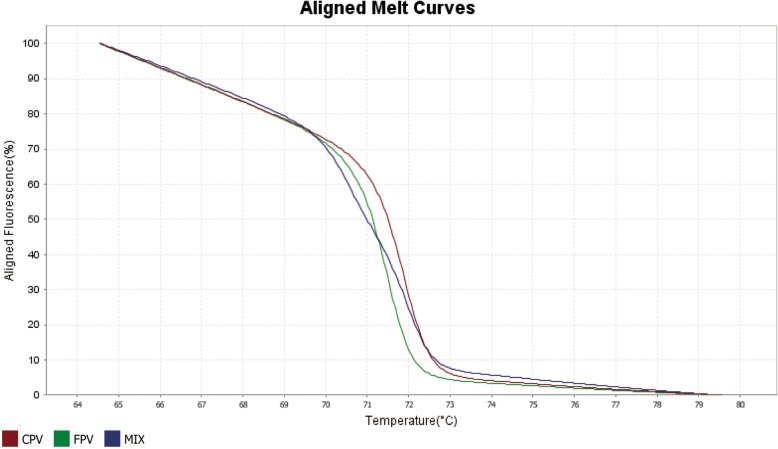
Discrimination between CPV and FPV by HRM analysis. FPV and CPV recombinant plasmid DNA, pEASY-FPV-H and pEASY-CPV-2a, were positive control in the qPCR-HRM analysis, and the mixtures of pEASY-FPV-H and pEASY-CPV-2a with different concentration ratios of 1:9 ~ 9:1 (v/v) were also amplified for detecting co-infection. The melt curve was acquired and analyzed by Applied Biosystems® High Resolution Melt Software v3.1. The results show aligned melt curve analysis of pEASY-FPV-H, pEASY-CPV-2a and the mixtures of the two standard plasmids at the ratio of 1:1 (v/v)

To assess the typing capacity of the designed primers, single plasmid and mixtures of pEASY-FPV-H and pEASY-CPV-2a were carried out in the qPCR-HRM assay. The FPV and CPV positive plasmid DNA samples generated melting curves. The different shape that HRM analysis software generated can distinguish FPV and CPV according to the specific melting temperature and shape of melting curves (Fig. 2). The Aligned melt curve plots revealed the melt curves as % melt (0–100%) over temperature. Aligned Melt Curves plot was generated by aligning the melt curves to the same fluorescence level using the pre- and post-melt regions.

### Analytical specificity and sensitivity of HRM assay

**Fig. 3:**
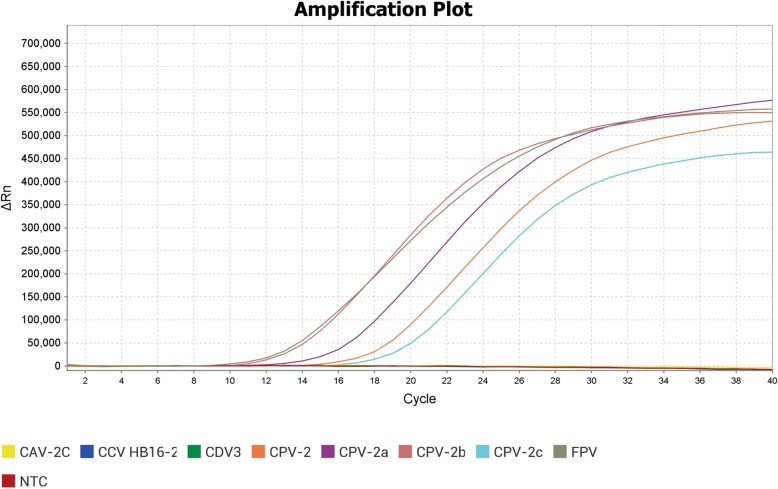
Analytical specificity of HRM assay. The results show HRM was specific for both CPV (the LN15–32 strain of CPV-2, the JL14–1 strain of CPV-2a, the BJ14–1 strain of CPV-2b and the BJ15–20 strain of CPV-2c) and FPV. No specific amplification was detected with other cat and dog viruses such as CDV, CCV, and CAV and negative for the sterile water control

Our results showed that the HRM assays were highly specific for LN15–32 strain of CPV-2, the JL14–1 strain of CPV-2a, the BJ14–1 strain of CPV-2b, the BJ15–20 strain of CPV-2c, and the commercial vaccine FPV (Fel-O-Vax-PCT), and that no non-specific fluorescence was caused by canine coronavirus (CCV), canine distemper virus (CDV), canine adenovirus (CAV) (Fig. 3), canine parainfluenza virus (CPIV), feline calicivirus (FCV), and feline herpesvirus-1 (FHV-1).

**Fig. 4:**
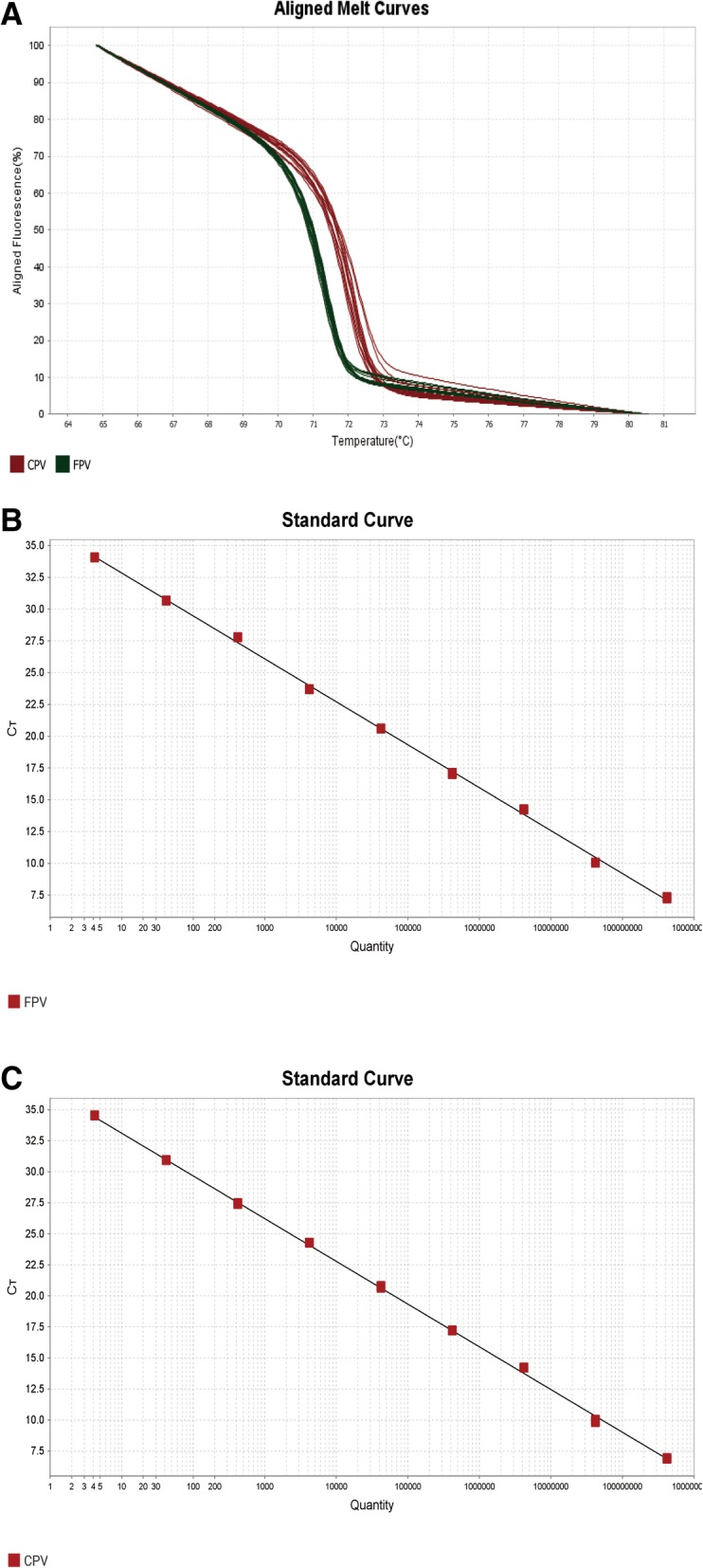
Analytical sensitivity of HRM assay. **a** Aligned Melt Curves of different concentrations of the plasmids. The two plasmids, diluted from 4.2 × 10^0^ to 4.2 × 10^8^ copies/μl, were amplified by qPCR and analyzed by HRM analysis software. The results showed FPV and CPV were successful distinguished in the dilution range. Standard curves obtained for FPV (**b**) and CPV (**c**) indicating the linearity and efficiency for detecting both viruses by qPCR. The dilutions of standard DNA are indicated on the x-axis, whereas the corresponding cycle threshold (Ct) values are presented on the y-axis. The assays were linear in the range of 4.2 × 10^0^ to 4.2 × 10^8^ template copies/μl, with a coefficient of determination (R^2^) of 0.999 for both FPV and CPV and reaction efficiency of 97.76 and 95.24%. The result showed that the limit detection of HRM assay is both 4.2 copies/μl for FPV and CPV

In order to evaluate sensitivity, a series of gradient dilutions of recombinant plasmid DNA, ranging from 4.2 × 10^0^ to 4.2 × 10^8^ copies/μl, were tested. The results showed FPV and CPV were successfully distinguished by the HRM assay (Fig. 4a). The detection limits of the HRM assay were 4.2 copies/μl of FPV and CPV plasmid DNA below 35 Ct (Fig. 4b, c). Besides, the standard curves of FPV and CPV showed a strong linear correlation between 10^0^ and 10^8^ copies/μl. Slope values were − 3.377 and − 3.442 for FPV and CPV, respectively, and R^2^ (coefficients of correlation reached values) was 0.999 for both viruses. Hence, the amount of CPV and FPV can be quantified based on those standard curves.

### Reproducibility of the HRM assay

The intra-assay and inter-assay reproducibility test indicated that the HRM was reproducible. The coefficient of variation of the HRM assay was between 0.5 and 3.4% in intra-assay and inter-assay, with three independent tests of CPV-2 and FPV at low (10^4^), medium (10^6^) and high concentration (10^8^) genome copies, as determined in triplicate.

### DNA sequencing

A total of 80 samples were identified using HRM based methods. Forty-two and 6 samples were positive for CPV and FPV, respectively. The results of the HRM showed that 2, 21, 8, and 11 samples were characterized as CPV types 2, 2a, 2b, and 2c, respectively, among the CPV positive samples. A total of 45 samples were successfully sequenced. Data analysis indicated that 39 samples were CPV positive, and 6 samples were FPV positive. The results showed the percentage of agreement, relative sensitivity and specificity between HRM and DNA sequencing were 96.25, 91.43 and 100%.

### Virus isolation

Of those 80 positive samples, virus isolation was successful in 36 samples. Those isolates were determined to be subgroup (32/36) CPV and (4/36) FPV based on sequence information. Compared with virus isolation, the percentage of agreement, relative specificity, and sensitivity of HRM assay were 78.5, 72.72, and 100%, respectively.

## Discussion

CPV and FPV are the contagious viral cause of enteritis in domestic dogs and cats as well as for various wild species [19]. And clinical diagnosis is always indecisive and slow, due to the fact that a lot of viral pathogens can cause similar clinical signs like diarrhea in cats such as CDV, coronavirus, adenovirus, and rotavirus. Likewise, diseases associated with FPV and CPV are concurrent [20], giving rise to the difficulty of specific pathogen detection of the pathogen. At present, various diagnostic methods were developed for detection of CPV and FPV, such as PCR, quartz crystal microbalance biosensor, hemagglutination, hemagglutination inhibition, immunochromatographic, enzyme-linked immunosorbent assay and so on [21–24]. However, the traditional diagnostic methods cannot detect the pathogen with complete accuracy and are time-consuming and expensive. Thus, the study developed a rapid, inexpensive, sensitive and accurate diagnostic method for discrimination of CPV and FPV with a single PCR.

HRM assay was recently reported using for genotyping and mutation scanning, which identify a single base change in short fragment up to 400 bp [25, 26]. A single base change from a purine to pyrimidine was easily identified, which results in a change in Tm of about 1 °C caused by changes of hydrogen bond number. However, a mutation of A/T or C/G is difficult to discern causing a small change of Tm of about 0.4 °C [27]. In this study, we designed a pair of primers targeted to amplify 101 bp segment which contains the SNP C4408A to specifically screen CPV and FPV. The data that the qPCR instrument generated were auto-analyzed in HRM analysis software. The HRM assay developed herein is an extremely sensitive, accurate technique to detect and differentiate the CPV and FPV variants. The detection limits of CPV and FPV were both 4.2 copies/μl (Fig. 4b and c). On the other hand, viral load can be determined by a standard curve. Although it’s not essential for illness diagnosis, quantitation of virus load is of the greatest usefulness in some respects of describing the relationship of viral load and disease progression by monitoring the change of viral titer during infection, surveying vaccine efficacy in vaccine production and assessing the effectiveness of therapy.

The results of detection and genotyping of 80 clinical samples with three methods showed a perfect agreement between HRM assay, DNA sequence, and virus isolation, with a percentage of agreement of the HRM assays and the other methods was 96.25 and 85%, respectively. Forty-eight samples were genotyped using HRM assay and 45 samples were identified using DNA sequence except for 3 samples. Of the 48 samples, 2 were classified as CPV-2, 21 were CPV-2a, 8 were CPV-2b, 11 were CPV-2c and 6 were FPV, respectively. And we found two CPV-2c isolates from cats and no other CPV types in cat samples. DNA sequencing and Virus isolations were all time-consuming and labor intensive, while the HRM assay has a lot of advantages over DNA sequencing, that can simultaneously detect many single-infection or co-infections in one assay, and data collection and analysis were automatic. This HRM assay was reproducible and can be used for genotyping CPV and FPV in clinical samples instead of DNA sequencing.

## Conclusion

In conclusion, our results indicate that the HRM assay can be used for clinical diagnosis and epidemiological surveillance of CPV and FPV mono infection or co-infection.

## Methods

### Viruses and cells

The viruses (LN15–32 strain of CPV-2, the JL14–1 strain of CPV-2a, the BJ14–1 strain of CPV-2b, the BJ15–20 strain of CPV-2c, HB16–2 strain of CCV, CDV3 strain of CDV, JL1 strain of CPIV, HB15–1 strain of FCV, and BJ16 strain of FHV-1) and the cells (F81, MDCK and Vero) were described in our previous study [28]. FPV (Fel-O-Vax-PCT) (Boehringer Ingelheim Vetmedica Inc., Missouri, USA) and CAV-2C (Jilin Teyan Biotechnology Co., Ltd. Changchun, China), two commercial vaccines, were also used in this study. The cells were grown in Dulbecco’s modified Eagle’s medium (DMEM) supplemented with 10% fetal calf serum in 5% CO_2_ at 37 °C.

### Samples

A total of 80 fresh fecal samples (58 from dogs and 32 from cats) were obtained using rectal swabs from Hebei and Jilin province of China. All the animals showed the clinical signs of diarrhea and so on. All the samples were obtained from privately owned animals via participating veterinary hospital. Collected swabs were immersed in 1 ml DMEM supplemented with 2000 U penicillin G/ml and 100 mg/ml streptomycin. DNA was extracted from 200 μl of the suspension using the MiniBest DNA/RNA viral extract kit ver 5.0 (Takara Biotechnology Co., Ltd., Dalian, China) following the manufacturer ‘s instructions. cDNA was obtained from 500 μl CCV HB16–2 and CDV3 suspension as described previously [29]. DNA and cDNA samples were stored at − 80 °C until use.

### Primer design

All full-length genomes of CPV and FPV were retrieved from GenBank and were aligned to find mutation using MEGA7.0 software package [30]. Two pairs of primers (F1/R1, F2/R2) were designed using the primer 5.0 software package [31], which amplify 361 and 101 bp products containing the SNP C4408A between the sequences of CPV and FPV. Alignment of all of the sequences of CPV and FPV from GenBank and we found the 4408 were conservative, where FPV and CPV present A and G, respectively. That SNP is responsible for synonymous mutations that had no effect on the amino acid Ala at residue 541 of the VP2 protein. These primers were used for constructing CPV and FPV standard plasmids and developing HRM-assay, respectively. Primers were synthesized by Comate Bioscience Company Limited (Changchun, China). Sequence, position and amplicon size of PCR and real-time PCR are shown in Table 1.

**Table 1:** Sequence, position and specificity of primers used in this study

Assay	Primer	Sequence 5’to 3’	Position^a^	Amplicon size
Standard DNA	F1	ATTATTTGTAAAAGTTGCGCC	4276–4296	361 bp
	R1	CTAAATCCTATATCAAATACAAGTACAA	4609–4636	
HRM assay	F2	TCAGATTTTTGGTGGAAAGGTAA	4358–4380	101 bp
	R2	TGGTTATCTACATTAATACTCATTTGTTG	4430–4458	

^a^Primer positions are referred to the sequence of CPV strain Laika-1993 (GenBank accession number: JN033694)

### Standard plasmid preparation

The target *VP2* gene of CPV and FPV was PCR amplified using 2 × Ex Taq Mix (Takara Biotechnology Co., Ltd., Dalian, China) with a pair of primers (F1/R1). The reaction was carried out in a total volume of 50 μl containing 2 × Ex Taq Mix, 10 μM primers and 5 μl of DNA. The thermal protocol was carried out as follows: initial denaturation at 95 °C for 5 min, 35 cycles at 95 °C for 30 s, annealing at 50 °C for 1 min, at 72 °C for 2 min, and a final extension at 72 °C for 10 min. The PCR products were purified by using PureLink® PCR Purification Kit (Invitrogen Life Technologies, California, USA), following the manufacturer’s instructions. The purified fragments were ligated separately into pEASY™-T5 zero vector and propagated in Trans 5α cell (Beijing TransGen Biotech Co., Ltd., Beijing, China), following the manufacturer’s instructions. Then, colony PCR was performed to select correct cloning using primers F1/R1. Plasmid DNA was purified using Trans Plasmin kit (Beijing TransGen Biotech Co., Ltd., Beijing, China), and was verified by 3% agarose gel electrophoresis and sequenced in Comate Bioscience Company Limited (Changchun, China). Subsequently, two positive plasmids were selected and named as pEASY-FPV-H and pEASY-CPV-2a, respectively, based on the DNA sequencing. The recombinant plasmid was stored in − 80 °C until use after quantifying using NanoPhotemeter® NP80 (Implen GmbH, Munich, Germany).

### qPCR and HRM analysis

The plasmid was amplified using the QuantStudio™ 3 Real-Time PCR System (ABIInc. CA, USA) with a volume of 30 μl per tube. Likewise, mixtures of pEASY-FPV-H and pEASY-CPV-2a at a ratio of 1:9 ~ 9:1 (v/v) were also amplified for detecting co-infection samples caused by FPV and CPV. Each tube contained 15 μl Applied Biosystems® MeltDoctor™ reagents (Life Technologies Corporation I Carlsbad, CA, USA), 0.5 μl of primer F2 and R2 with a concentration of 10 M, 1 μl DNA, and 13 μl ddH_2_O. Three replicates per sample and negative control were set. The cycling conditions followed as 50 °C for 2 min and 95 °C for 10 min, PCR stage: 40 cycles at 95 °C for 15 s and 58 °C for 40 s, melt curve: 95 °C for 15 s, 60 °C for 1 min to 95 °C for 15 s at a rate of 0.025 °C/s. The database was further analyzed using the Applied Biosystems® High Resolution Melt Software v3.1 (Thermo Fisher Scientific Inc. CA, USA). The software automatically assigned samples to one variant with a similar shape of control by comparing the melt curves of the samples against those generated from positive control (standard DNA). The results can be plotted in various ways, including raw melt curves, aligned melt curves and difference plot, according to the melting behavior of their amplicons determined using the High Resolution Melt software v3.1.

### Analytical specificity and sensitivity of HRM assay

To exclude cross-reactivities between CPV/FPV and other canine viral pathogens, the DNA or cDNA of CAV-2C, CCV HB16–2, FPV (Fel-O-Vax-PCT), CPV-2/2a/2b/2c and CDV3, were tested using HRM assay. The ddH_2_O was set as the negative control (NTC).

In order to assess the limit of detection of HRM assay, 10-fold serial dilutions of the plasmid DNA, ranging from 4.2 × 10^0^ ~ 4.2 × 10^8^ copies/μl, were used as templates to be amplified by the real-time PCR.

### Reproducibility of the HRM assay

Inter-assay and intra-assay reproducibility tests were performed in triplicate by testing three different titers of cell cultures infected with LN15–32 and FPV (Fel-O-Vax-PCT) commercial vaccine to evaluate the reproducibility of the HRM assay [28].

### DNA sequencing

To assess the accuracy of genotyping in HRM assay, PCR products of 48 fecal samples were sequenced using a pair of primer F1/R1 and aligned by MEGA7.0 software package.

### Virus isolation

Virus isolation was performed on the clinical swab samples using F81 cells for parvovirus as described previously [32]. Isolated viruses were identified by PCR assays [5], and genotyped based on sequencing and alignment using the MEGA7.0 software package.

## Abbreviations

CAV: Canine adenovirus
CCV: Canine coronavirus
CDV: Canine distemper virus
CPV: Canine parvovirus
FPV: Feline parvovirus
HI: Hemagglutination inhibition assay
HRM: High resolution melting
MGB: Minor groove binder probe
qPCR: Quantitative Polymerase Chain Reaction
SNP: Single Nucleotide Polymorphism
